# 

**Published:** 1842-08-06

**Authors:** 


**CONTENTS OF No. 32.**

BIBLIOGRAPHICAL NOTICES: Quarterly Summary of the Transactions of the College of Physicians of Philadelphia, for May, June, and July, - - - - - - - - - - - - 497
The Principles and Practice of Modern Surgery. By Robert Druitt. From the second London Edition. Illustrated with 50 wood engravings. With Notes and Comments by Joshua B. Flint, M.D., M.M.S.S., Lecturer on Therapeutic and Operative Surgery in the Louisville Academy of Medicine, &c. Philadelphia, 1842. - - - - - - - - - 505
Dr. M. W. Wilson's Clinical Reports:
Typhus Fever, - - - - - - - - - - 506
EDITORIAL: Medical Reform, - - - - - - - - - 507
Officers of the College of Physicians of Philadelphia, - - - - 508
ANALECTA: Modification of the Muscular Flap Operation for Amputation below the Knee-joint, - - - - - - - - 508
Complete Prolapsus and Separation of the Vagina - - 510
Injury to the Cartilages of the Ribs - - - - - - 510
Dr. Corrigan's case of Urea secreted in large quantity by the Peritoneum in a case of Ascites - - - - - - 511
M. Rossi's case of Recovery after Extirpation of the Uterus immediately after delivery - - - - - - - 512
M. Longet on the influence of the Nerves on Muscular Irritability - - - - - - - - - - - - 512

